# Late recurrent urothelial carcinoma in the Studer neobladder: conversion to continent reservoir

**DOI:** 10.3332/ecancer.2012.268

**Published:** 2012-09-13

**Authors:** AF Kotb, M Alkosiry, N AbdElkawy, MA Atta

**Affiliations:** 1 Faculty of Medicine, Alexandria University, Al-Kartoum Square, Alexandria, Egypt

**Keywords:** *neobladder*, *late recurrence*, *radical cystectomy*

## Abstract

Bladder cancer represents a considerable issue in Egypt and the Middle East. Radical cystectomy and orthotopic neobladder represent the standard of care for managing cases with invasive bladder tumour. There are few cases reported in the literature considering the urothelial recurrence in the urethra, connected to neobladder. We are presenting a rare case of a young female patient, with an aggressive urothelial tumour, recurring 13-year post-radical cystectomy, and the Studer neobladder. Our case was managed by urethrectomy and conversion of the neobladder into continent reservoir, with good short-term oncological and functional outcomes. We can conclude that bladder cancer cases should be followed thoroughly throughout their life. Follow-up urethroscopy and cytology should be done for all cases of post-radical cystectomy, regardless of patients’ symptoms.

**Key message:**

Late urothelial recurrence of post-radical cystectomy is possible and, in our case, happened 13 years following surgery. The Studer neobladder can be safely converted into continent reservoir, allowing good functional outcomes. Also, recurrence in the Studer neobladder can be safely managed, allowing good oncological outcomes, without the need for any ureteroileal interventions.

## Introduction

Carcinoma of the urinary bladder, being the second most common genitourinary malignancy, is considered as a major health problem in Egypt and the Middle East [1, 2].

Radical cystectomy is the gold standard treatment for patients with muscle invasive bladder cancer, with 5 years overall survival rate of 50–70% [3]. Orthotopic neobladder substitution is, currently, recommended for suitable surgical candidates.

Data in the literature are sparse, and risk factors are not well identified due to the low number of such cases [2, 4–7]. Most cases of urethral recurrence present within the first 24 months and rarely within 36 months [8, 9]. Our case is unique because it presents an aggressive urothelial tumour, with squamoid differentiation, initially diagnosed while the patient was 45 years old. The tumour did not show any recurrence during 10 years post-surgical management. Locally advanced urothelial recurrence was found 13-year post-radical cystectomy, and the pathological study showed the same initial specimen pathological findings.

## Case report

**Figure 1.**
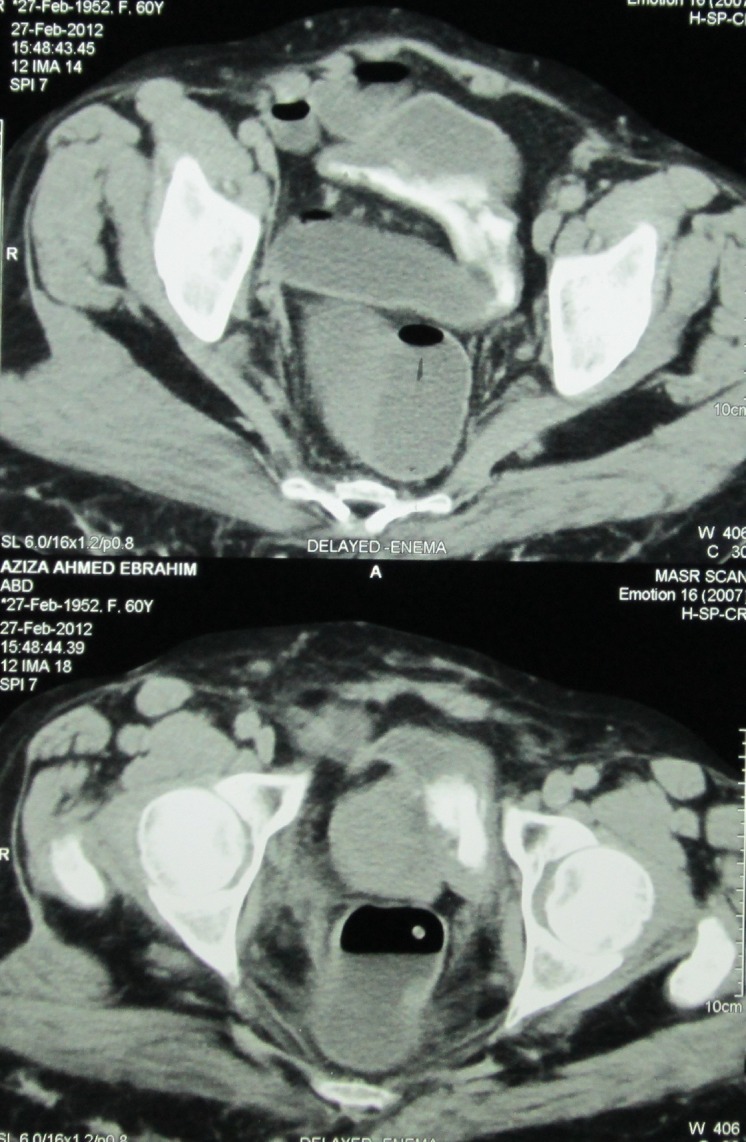
CT findings

A 59-year-old female patient presented to the urology outpatient clinic of the Faculty of Medicine, Alexandria University, complaining of recurrent attacks of frank haematuria. The case had a history of muscle invasive bladder urothelial tumour that was managed 13 years ago by anterior pelvic exenteration and the Studer neobladder. The pathology report, at that time, revealed T2 transitional cell carcinoma (TCC) with squamoid differentiation, high-grade disease with no associated carcinoma in situ (CIS). All lymph nodes removed were reactive and free of malignancy. The pathology report of the preceding cystoscopy was showing the same histology, and the tumour was large, single, nodular, and away from the trigon. The patient has been followed on an annual basis by laboratory and imaging studies, which were all free till 10 years following surgery. No endoscopic or cytology follow-up studies were done. This follow-up was stopped after 10 years, then the patient presented to our outpatient clinic 3 years later complaining of recurrent attacks of haematuria. Multi-phasic CT abdomen and pelvis revealed normal kidneys and a recurrent mass in the neobladder. No lymphadenopathy was detected by CT. Figure 1 shows the CT findings. Chest CT was free of metastases. The patient was admitted for endoscopic assessment.

During cystourethroscopy, examination under anaesthesia revealed a hard mobile mass involving the urethra and distal posterior wall of the pouch. Tumour tissues were found visibly infiltrating the vaginal wall. Endoscopy showed a non-papillary tumour with necrotic surface, infiltrating the posterior wall of the urethra and extending into the distal posterior wall of the neobladder. Resection was done using monopolar current, and the pathological report documented invasive TCC of the whole resected tumour. The patient was counselled and prepared for surgery.

Low-midline incision was done, overlapping the previous scar. A large Studer pouch was easily identified. The anterior wall of the pouch was opened in its proximal portion. Combined abdominal and vaginal exploration on the mass was done, removing the whole urethra, vagina, and the distal part of the pouch. Frozen section of the part related to the remaining pouch was done and was free of malignancy.

**Figure 2.**
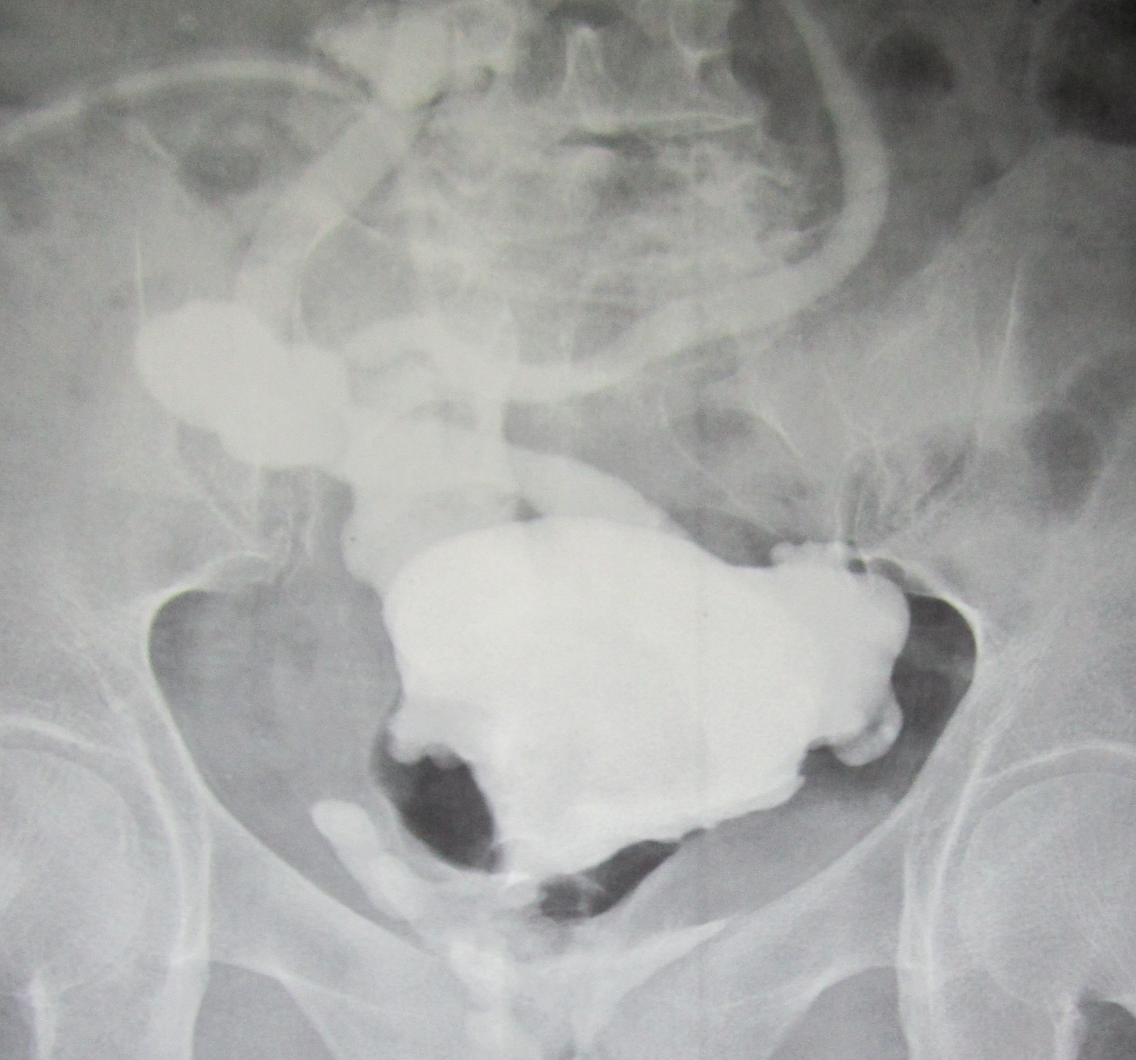
Pouchogram performed three weeks following surgery.

The remaining pouch was of a good volume, so we did not need to do augmentation. A spiral flap was taken from the pouch and directed upward toward the umbilicus. The remaining pouch was closed. The flap was formed as a tube over 14-Fr silicone catheter and pulled into the umbilicus to be a catheterizable stoma, so converting the Studer neobladder into continent reservoir. The proximal part of the catheterizable tube was intussuscepted into the pouch, to help making the patient continent. We did not try to look for the ureters and we did not put any ureteric stents to divert the urine. Pathology report confirmed the diagnosis of T3 TCC with negative surgical margins. The only post-operative encountered problem was low-serum albumin that was only slightly improving with daily plasma transfusion. Oral diet was resumed on the second post-operative day. Two weeks after surgery, the silicon catheter was removed. The stoma was continent, with no urine leak through it. The patient was easily catheterizing herself every 4 h, draining adequate amount of urine. However, she was complaining of urine leak per vagina. Pouchogram was done documenting the urine leak. Figure 2 shows the pouchogram done 3 weeks following surgery.

The silicon catheter was then reinserted and kept in place further for one week, allowing the patient to be dry. The last follow-up of the patient is 3 months following surgery, showing adequate healing and easily catheterizable umbilical stoma, with no urine leak. Close follow-up will be going on for the possibility of recurrence.

## Discussion

Late local secondary TCC, following radical cystectomy and ileal neobladder, is not well understood. Data in the literature are sparse, and risk factors are not well identified due to the low number of such cases. Stein *et al* [4] reported a low-urethral recurrence rate in 2% of 841 women managed by radical cystectomy and orthotopic neobladder, over a follow-up, exceeding 20 years. Badawy *et al* [2] studied 78 women managed by ileal neobladder, over a mean follow-up of five years and could detect urethral recurrence in 1.2% of cases. Jentzmik *et al* [5] reported urethral recurrence in 0.8% of women, over a median follow-up of five years.

Stein *et al* [6] studied the risk factors for recurrence in 768 men, managed by radical cystectomy. They could conclude that prostatic involvement and the type of urinary diversion are the two independent risk factors associated with recurrence. Hassan *et al* [7] studied recurrence in 54 female patients managed by radical cystectomy, over a median follow-up of less than three years. They could find that bladder neck involvement and stomal diversion were the risk factors associated with recurrence.

In Alexandria University, we prefer not to do any stomal diversion to our female patients, for social reasons. We either do orthotopic ileal neobladder or the modified ureterosigmoidostomy, doing the technique published by ourselves in 1996 [10]. We did not routinely investigate the urethra in asymptomatic patients and we currently believe that we should establish routine urethral investigations, through urethral wash and urethroscopy, that can be currently done easily as an outpatient procedure, with the availability of flexible instruments.

Treatment of the recurrent tumour in the neobladder depends on the tumour size, multi-focality, pouch volume, and the technique of ileal neobladder initially done. The Studer neobladder with a multifocal tumour may be managed by urethrectomy and excision of the neobladder, with conversion of the isoperistaltic limb into ileal conduit. Small urethral recurrence can be managed with urethrectomy and conversion into continent reservoir, with or without augmentation, depending on the remaining pouch volume.

Our case was initially managed by the Studer ileal neobladder post-radical cystectomy. Managing the tumour recurrence, we performed urethrectomy, including the attached invaded part of the vagina and removing the distal part of the pouch, until the tumour-free edge was confirmed by the frozen section pathology. The remaining pouch seemed to be of adequate volume and we did not prefer doing augmentation of the remaining pouch, due to the low-performance status of the patient and suboptimal liver functions that made healing an important issue to consider. We did convert the neobladder into a continent reservoir. The patient had a smooth follow-up. At a three month follow-up, the patient was in a good shape, had no urine leak from stoma or vagina, and the stoma was easily catheterizable every 4 h.

EAU guidelines defined urine cytology following radical cystectomy as an optional measure and to be done according to the patient risk criteria. Clark *et al* [11] retrospectively reviewed 1,054 patients and recommended screening cytology, in the follow-up of such patients. In our institutions, we were not doing screening cytology or any endoscopic study, unless the patient had a complaint, as in the way our case was managed. We believe that we may have to do such screening for all patients, post-radical cystectomy. Also we believe that we may have another group of patients that may have developed asymptomatic small recurrence and may need further surgical care.

## Conclusion

Late urethral recurrence of post-radical cystectomy and orthotropic neobladder is possible and warrants continuous urethral screening for the rest of the patient’s life. Conversion of the Studer neobladder into continent reservoir is a practical easy operation, associated with good functional and oncological outcomes.
